# Ceramide kinase-mediated C1P metabolism attenuates acute liver injury by inhibiting the interaction between KEAP1 and NRF2

**DOI:** 10.1038/s12276-024-01203-4

**Published:** 2024-04-01

**Authors:** Wei Dong, Qing Li, Xing Lu, Jianfeng Lan, Zhidong Qiu, Xuehong Wang, Junnan Wang, Xiaojiao Zheng, Sifan Chen, Chong Zhang, Junfei Jin

**Affiliations:** 1 Guangxi Key Laboratory of Molecular Medicine in Liver Injury and Repair, the Affiliated Hospital of Guilin Medical University, Guilin, Guangxi China; 2 Department of Hepatobiliary and Pancreatic Surgery, the First Affiliated Hospital of Zhengzhou University, Zhengzhou, Henan China; 3 Xiangya Hospital, Central South University, Changsha, Hunan China; 4 Guangxi Health Commission Key Laboratory of Basic Research in Sphingolipid Metabolism Related Diseases, the Affiliated Hospital of Guilin Medical University, Guilin, Guangxi China; 5 China-USA Lipids in Health and Disease Research Center, Guilin Medical University, Guilin, Guangxi China; 6 Center for Translational Medicine and Shanghai Key Laboratory of Diabetes Mellitus, Shanghai Sixth People’s Hospital Affiliated to Shanghai Jiao Tong University School of Medicine, Shanghai, China; 7 Guangdong Provincial Key Laboratory of Malignant Tumor Epigenetics and Gene Regulation, Guangdong-Hong Kong Joint Laboratory for RNA Medicine, Medical Research Center, Sun Yat-Sen Memorial Hospital, Sun Yat-Sen University, Guangzhou, Guangdong China; 8 Nanhai Translational Innovation Center of Precision Immunology, Sun Yat-Sen Memorial Hospital, Foshan, Guangdong China

**Keywords:** Mechanisms of disease, Homeostasis

## Abstract

Acute liver injury is the basis of the pathogenesis of diverse liver diseases. However, the mechanism underlying liver injury is complex and not completely understood. In our study, we revealed that CERK, which phosphorylates ceramide to produce ceramide-1-phosphate (C1P), was the sphingolipid pathway-related protein that had the most significantly upregulated expression during acute liver injury. A functional study confirmed that CERK and C1P attenuate hepatic injury both in vitro and in vivo through antioxidant effects. Mechanistic studies have shown that CERK and C1P positively regulate the protein expression of NRF2, which is a crucial protein that helps maintain redox homeostasis. Furthermore, our results indicated that C1P disrupted the interaction between NRF2 and KEAP1 by competitively binding to KEAP1, which allowed for the nuclear translocation of NRF2. In addition, pull-down assays and molecular docking analyses revealed that C1P binds to the DGR domain of KEAP1, which allows it to maintain its interaction with NRF2. Importantly, these findings were verified in human primary hepatocytes and a mouse model of hepatic ischemia‒reperfusion injury. Taken together, our findings demonstrated that CERK-mediated C1P metabolism attenuates acute liver injury via the binding of C1P to the DGR domain of KEAP1 and subsequently the release and nuclear translocation of NRF2, which activates the transcription of cytoprotective and antioxidant genes. Our study suggested that the upregulation of CERK and C1P expression may serve as a potential antioxidant strategy to alleviate acute liver injury.

## NRF2 activation: C1P’s path to redox homeostasis success

Liver diseases are a major worldwide health problem, and the exact causes of sudden liver damage are not completely known. Scientists discovered that a protein called Ceramide Kinase (CERK) and its product Ceramide-1-Phosphate (C1P) are vital in safeguarding the liver from harm. The research, led by Wei Dong and his team, revealed that CERK and C1P trigger a crucial protective process in the liver, known as the NRF2-KEAP1 signaling pathway. This process helps keep a balance of oxidation and reduction reactions in the liver, shielding it from harm. The scientists discovered that C1P attaches to a protein named KEAP1, which enables another protein, NRF2, to enter the cell’s core and trigger the protective process. This study could pave the way for new liver disease treatments.

This summary was initially drafted using artificial intelligence, then revised and fact-checked by the author.

## Introduction

In recent decades, liver diseases have become a major health problem worldwide, causing 1.96 million deaths per year^1^. Acute liver injury is a fundamental process in many liver diseases, including nonalcoholic or alcoholic steatohepatitis, viral hepatitis, cholestasis, and drug-induced liver injury. However, the molecular mechanisms underlying acute liver injury are poorly understood.

Oxidative damage, which is a major driver of tissue degeneration and pathology, has been associated with diseases ranging from Alzheimer’s disease to liver injury^2,3^. As the liver performs a broad spectrum of metabolic functions via oxidation and reduction reactions, the liver is the major organ in which active redox reactions and reactive oxygen species (ROS) attacks occur^4^. The pivotal role of oxidative damage in acute liver injury has attracted increasing amounts of attention from researchers^5,6^. NRF2-KEAP1 signaling is a crucial inducible mechanism that plays a role in maintaining redox homeostasis. Under homeostatic conditions, KEAP1 binds to NRF2, which is a nuclear transcription factor with antioxidant effects, sequesters NRF2 in the cytosol, and promotes proteasomal degradation of NRF2^7^. In response to oxidative stress, cysteine residues in KEAP1 are oxidized, causing a conformational change that disrupts its interaction with NRF2 and leads to the release of NRF2. Once released, NRF2 escapes KEAP1-mediated degradation and translocates to the nucleus, where it promotes the transcription of antioxidant genes, such as NQO1 and HO-1^8,9^.

Oxidative damage is a sophisticated process that is characterized by an imbalance between antioxidant capacity and ROS production and is involved in the dysfunction of subcellular structures or organelles, such as the plasma membrane, endoplasmic reticulum, peroxisomes, and especially mitochondria^10^. As sphingolipid (SPL) is an important determinant of the biophysical properties of membranes and a regulator of mitochondrial dynamics, a growing body of research has shown that disruption of SPL metabolism is associated with oxidative injury^11^. The SPL metabolism signaling pathway includes many bioactive sphingolipid metabolites that are powerful signaling molecules, such as ceramide, C1P, and S1P.

Ceramide is phosphorylated by CERK to form ceramide-1-phosphate (C1P). Ceramide kinase-like protein (CERKL) is the only known homolog of CERK, but multiple studies have shown that CERKL is not involved in C1P metabolism^12,13^. Multiple studies have demonstrated the antioxidative effects of CERKL^14–16^, but the relationship between CERK and oxidative damage is unclear. Interestingly, the level of CERK transcription was associated with oxidative stress in a NAFLD mouse model, which suggested that CERK might be involved in oxidative stress in the liver^17^.

Both SPL metabolism and the NRF2-KEAP1 signaling pathway are crucial for regulating redox homeostasis. Several studies have reported that SPL metabolism has a strong but complex association with the NRF2 signaling pathway^18–23^, but little is known about the relationship between CERK/C1P and the NRF2 signaling pathway. In our study, we elucidated the relationship between C1P metabolism and NRF2 signaling pathway activation and discovered that C1P increased the level of NRF2 protein by binding to the DGR domain of KEAP1 and subsequently releasing NRF2.

## Materials and methods

### Pulldown assay to assess sphingolipid bead–protein interactions

Ceramide-coated beads and C1P-coated beads were purchased from Echelon Biosciences. Briefly, 100 μL of ceramide-coated beads, C1P-coated beads, or control beads were washed five times with washing buffer (10 mM HEPES, pH 7.4; 150 mM NaCl; 0.5% NP-40) on ice. Cells from the control groups and treatment groups were lysed with the appropriate NP-40 lysis buffer (Beyotime, P0013F) and allowed to homogenize on ice for 10 min. The samples were subsequently centrifuged at 13,400×*g* for 10 min at 4 °C. The supernatants were collected and incubated with the washed beads on a rotary shaker at 4 °C overnight. Then, the beads were washed with washing buffer five times on ice. The supernatants were discarded carefully, and the beads were resuspended in 40 μL of 2× SDS loading buffer and boiled (100 °C) for 5 min. Finally, the supernatants were collected and used as samples to analyze target proteins. KEAP1 and NRF2 expression was measured via western blotting.

### Coimmunoprecipitation (Co-IP)

Briefly, cells from the control groups and treatment groups were lysed with the appropriate cell lysis buffer for western blotting and IP (Beyotime, P0013) on ice for 10 min. Then, the samples were centrifuged at 13,400×*g* for 10 min at 4 °C. The supernatants were collected and incubated with 8 μg of antibody or control IgG on a rotary shaker at 4 °C overnight to form immune complexes. Forty microlitres of protein A/G-magnetic beads were washed three times with cell lysis buffer for western blotting and IP, added to the above immunocomplexes, and incubated for 8 h on a rotary shaker at 4 °C. Then, the magnetic beads were separated by a magnetic separator and rinsed with prechilled cell lysis buffer five times on ice. The pellet was resuspended in 40 μL of 2× SDS loading buffer, mixed well by vortexing, and boiled (100 °C) for 5 min. Finally, the supernatants were collected and used as samples to analyze target proteins. The levels of KEAP1, NRF2, and ubiquitinated proteins were measured via western blotting.

### Molecular docking

The crystal structure file of mouse KEAP1 was obtained from the RCSB Protein Data Bank (http://www.rcsb.org/pdb). The Protein Data Bank code was 7C60. The structure of C1P (d18:1/18:0) was collected from PubChem (PubChem CID: 5283583). Molecular docking was performed with AutoDock Vina 1.1.2. The docking experiments were performed with the default parameters of AutoDock Vina^24,25^.

### Statistical analysis

Analyses were performed using GraphPad Prism 8 (GraphPad, Inc., USA). Student’s *t* test or analysis of variance (ANOVA) was used to analyze the statistical significance of the differences. Statistical differences between two groups were calculated by the *t* test. *P* values were corrected for multiple testing according to Dunnett’s (when we compared several groups with a single control group) or Tukey’s (when we compared each other group) post hoc tests. When two parameters of two different groups were compared, two-way ANOVA was performed. *P* < 0.05 was considered to indicate statistical significance.

Further details of the materials and methods are provided in the Supplementary Materials and Methods section.

## Results

### High expression of CERK plays a role in CCl_4_-induced acute liver injury

**Fig. 1:**
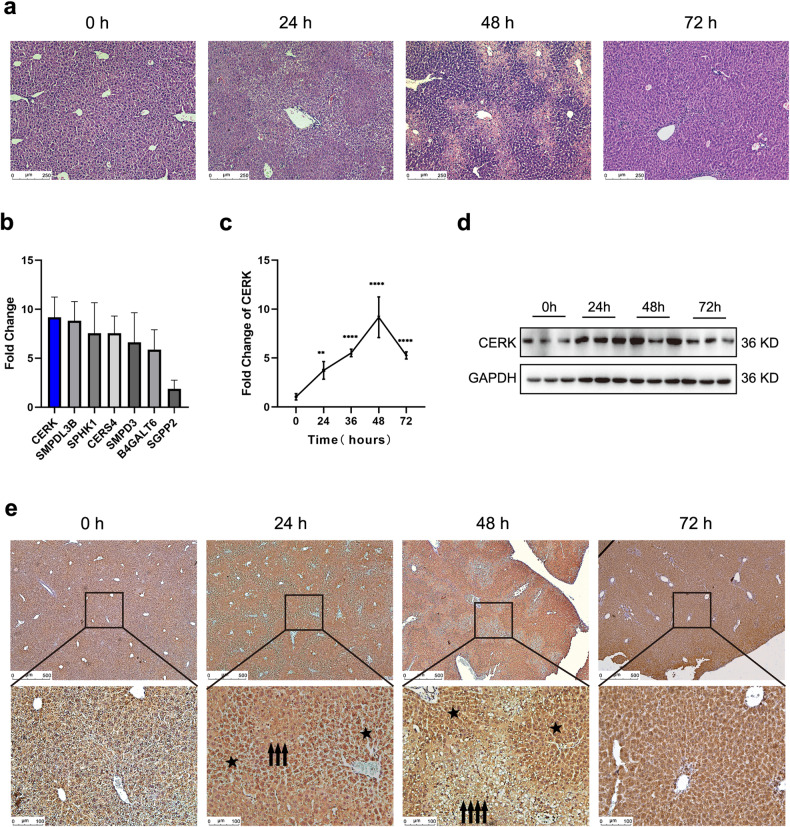
High expression of CERK is involved in CCl_4_-induced acute liver injury. **a** Representative images of liver injury sites stained with hematoxylin and eosin (H&E) are shown. Scale bar: 250 μm. **b** qRT‒PCR analysis of the seven top genes involved in sphingolipid metabolism (fold change>6) in the validation groups was performed. (*n* = 5/group). **c** qRT‒PCR analysis of CERK expression was performed in acute liver injury time course experiments. (*n* = 5/group). **d** The protein expression of CERK in acute liver injury time course experiments was determined by western blotting. **e** Representative images of IHC staining for CERK in tissues at different time points after acute liver injury are shown. The arrow indicates severely injured hepatocytes, and the asterisk indicates mildly injured hepatocytes. Scale bar: 500 μm (top), 100 μm (bottom). One-way ANOVA was used to analyze the significant differences. **P< 0.01, ***P< 0.001, and ****P < 0.0001.

A model of acute liver injury was established by a single injection of CCl_4_ (1 ml/kg) into 8- to 10-week-old mice. To determine the optimal time point for subsequent study, the severity of hepatic injury at different time points after CCl_4_ administration was evaluated by histopathological staining and serum biochemistry analyses, including AST, ALT, and LDH measurements. CCl_4_-induced liver injury was confirmed based on hepatocyte necrosis (Fig. 1a) and increased hepatic enzyme levels (Supplementary Fig. 1a–c), and the time course experiments suggested that liver injury was most severe at 48 h after CCl_4_ administration; thus, subsequent experiments related to acute liver injury were performed at these time points. Using a PCR array, we found that seven genes in the sphingolipid pathway showed the most significantly upregulated expression (fold change >6) in the livers of mice administered CCl_4_ after 48 h compared to those of normal mice (Supplementary Fig. 2). To validate this result from the PCR array, we also measured the expression of these differentially expressed genes in multiple samples and found that the upregulation of ceramide kinase (CERK) expression was the most obvious (Fig. 1b). Therefore, CERK was selected for subsequent study. Upon CCl_4_ treatment, CERK mRNA (Fig. 1c) and protein (Fig. 1d) expression in the liver gradually increased, peaked at 48 h, and then decreased. Interestingly, a stronger intensity of CERK staining was observed in mildly injured hepatocytes (asterisk) than in necrotic hepatocytes (arrow), as revealed by immunohistochemistry (Fig. 1e), suggesting that the upregulation of CERK expression might protect against acute liver injury.

### CERK inhibition exacerbates liver injury in vivo and in vitro

**Fig. 2:**
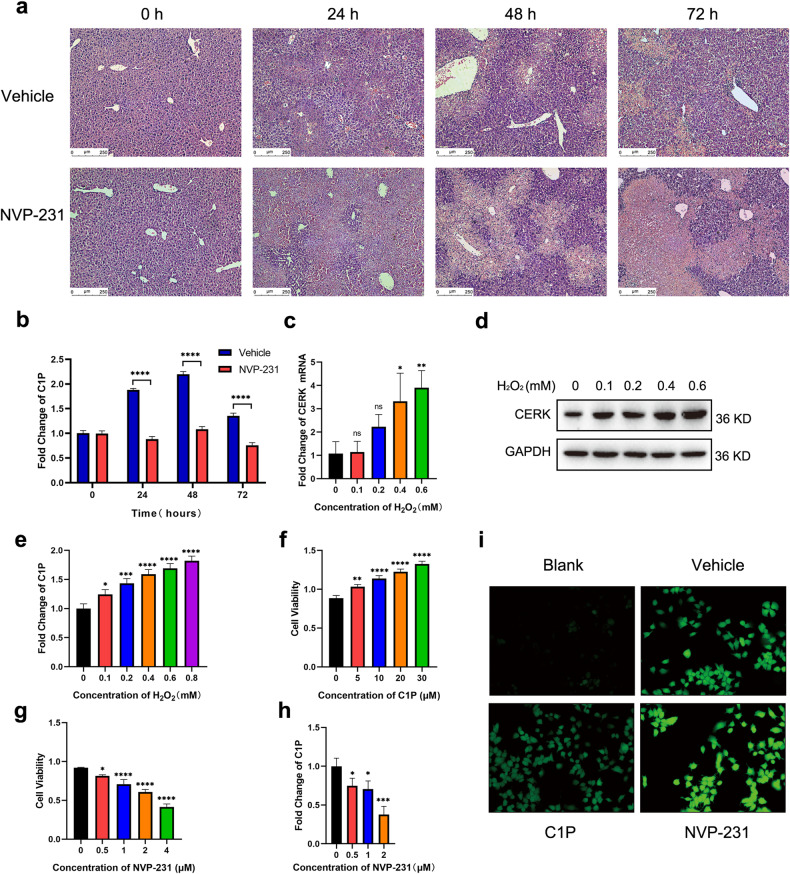
CERK inhibition exacerbates liver injury in vivo and in vitro. **a** Representative images of liver injury sites stained with hematoxylin and eosin (H&E) are shown. Scale bar: 250 μm. **b** The relative concentration of total C1P in acute liver injury time course experiments induced by CERK inhibition was measured via ELISA (*n* = 5/group). **c** The mRNA expression of CERK in AML12 cells with H_2_O_2_-induced oxidative damage was measured by qRT‒PCR (*n* = 3/group). **d** The protein expression of CERK in AML12 cells with H_2_O_2_-induced oxidative damage was measured via WB. **e** The relative concentration of total C1P in AML12 cells with H_2_O_2_-induced oxidative damage was measured via ELISA (*n* = 3/group). **f** The viability of AML12 cells treated with C1P for 24 h and exposed to H_2_O_2_ (0.4 mM) was determined (*n* = 3/group). **g** The viability of AML12 cells treated with NVP-231 for 24 h and exposed to H_2_O_2_ (0.4 mM) was determined (*n* = 3/group). **h** The relative concentrations of total C1P in AML12 cells treated with NVP-231 for 24 h were measured via ELISA following exposure to H_2_O_2_ (*n* = 3/group). **i** The intracellular ROS level was measured by an ROS assay kit. H_2_O_2_ was added to 20 μM C1P and 2 μM NVP-231 for 24 h, while the same volume of vehicle was added to the vehicle group. **b** Two-way ANOVA; **c**, **e**–**h** one-way ANOVA. *P < 0.05, **P < 0.01, ***P < 0.001, ****P < 0.0001, and ns not significant.

To further investigate the function of CERK in acute liver injury, CERK activity was inhibited using NVP-231, which affects C1P production but not CERK expression. In vivo, time course experiments suggested that NVP-231 administration exacerbated liver injury after CCl_4_ treatment, as indicated by the increase in ALT, AST, LDH levels (Supplementary Fig. 3a–f) and necrotic areas (Fig. 2a and Supplementary Fig. 4) at early time points. As expected, NVP-231 blocked the increase in C1P levels after CCl_4_ administration (Fig. 2b). To rule out the possibility that the NVP-231-mediated worsening of liver injury was caused by NVP-231 toxicity, mice were administered NVP-231 alone. As expected, hepatotoxicity was not observed when treated with NVP-231 alone, as shown by the absence of hepatocellular necrosis (Supplementary Fig. 5a) and the absence of a significant increase in serum ALT, AST, and LDH levels (Supplementary Fig. 5b–d).

**Fig. 3:**
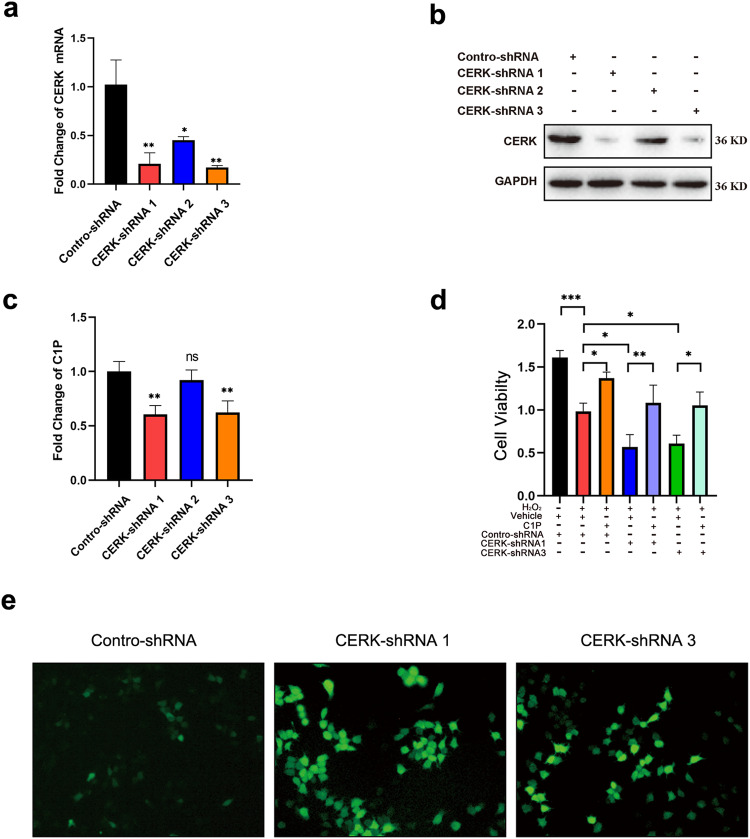
CERK knockdown exacerbated acute liver injury in vitro. **a** The mRNA expression of CERK in AML12 cells with stable CERK knockdown was measured by qRT‒PCR. **b** The protein expression of CERK in AML12 cells with stable CERK knockdown was measured by western blotting. **c** The relative concentrations of total C1P in AML12 cells with stable CERK knockdown were measured via ELISA. **d** The viability of stable CERK-knockdown AML12 cells treated with C1P. **e** The level of intracellular ROS in AML12 cells with stable CERK knockdown was measured via an ROS assay kit. **a**, **c** Student’s *t* test; **d** one-way ANOVA. *P < 0.05, **P < 0.01, ***P < 0.001, and ns not significant.

To investigate the underlying mechanisms, H_2_O_2_ was used to induce hepatocyte damage in vitro to mimic CCl_4_-induced liver injury in vivo. First, H_2_O_2_ treatment caused a significant decrease in cell viability (Supplementary Fig. 6a), which was accompanied by an increase in the expression of the antioxidative enzyme CAT and oxidative damage indicators such as MDA and ROS in a concentration-dependent manner (Supplementary Fig. 6b–d). Second, we found that the mRNA and protein expression of CERK was upregulated in a concentration-dependent manner (Fig. 2c, d), which was accompanied by an increase in C1P (Fig. 2e). Third, AML12 cells were treated with different concentrations of C1P or NVP-231 for 24 h in the presence of H_2_O_2_. C1P treatment increased cell viability (Fig. 2f), while NVP-231 decreased cell viability (Fig. 2g). NVP-231 reduced C1P levels in a dose-dependent manner (Fig. 2h). C1P treatment reduced ROS production, while ROS accumulation was found in the NVP-231 group (Fig. 2i). Decreased MDA levels and increased CAT activity were observed in the C1P group, and these changes were reversed in the NVP-231 group (Supplementary Fig. 7a, b). Additionally, C1P or NVP-231 alone did not affect cell viability in the absence of H_2_O_2_ (Supplementary Fig. 8a, b). Fourth, AML12 cells with stable CERK knockdown were generated (Fig. 3a–c). In the presence of H_2_O_2_, CERK knockdown reduced cell viability, and C1P addition rescued the loss of cell viability in CERK-silenced cells (Fig. 3d). CERK knockdown elevated ROS levels (Fig. 3e) and MDA levels while reducing CAT activity (Supplementary Fig. 9a, b). These findings suggested that C1P acted as an antioxidant and exerted cytoprotective effects under oxidative stress conditions.

### CERK/C1P protects against oxidative damage via upregulation of NRF2 protein expression

**Fig. 4:**
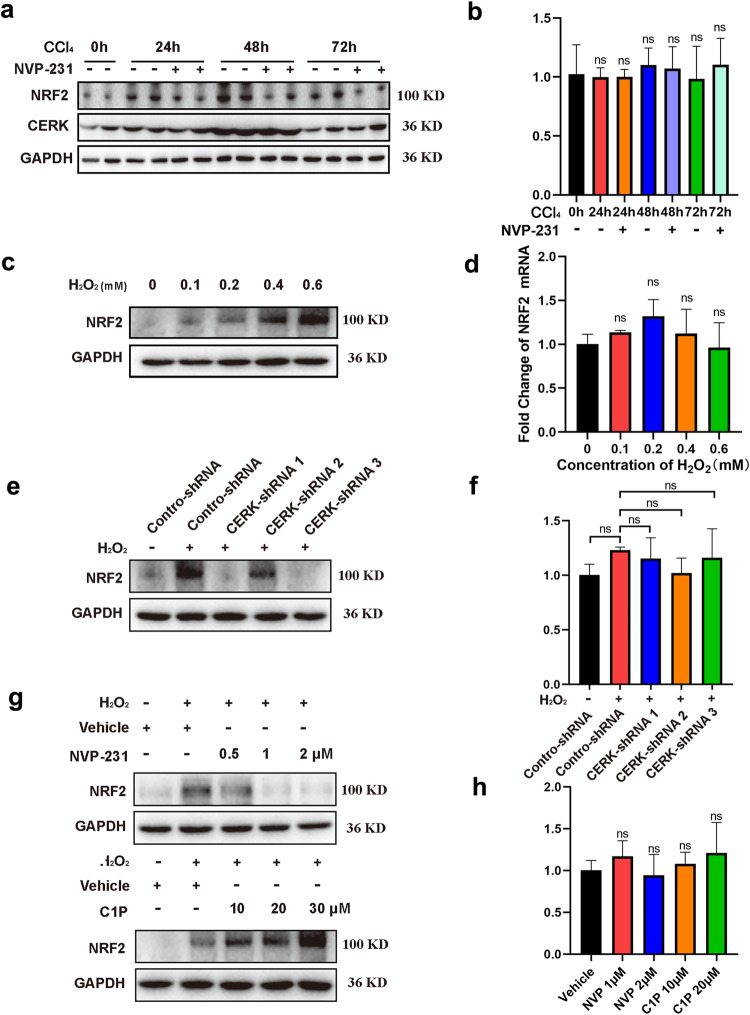
CERK/C1P protects against oxidative damage via the upregulation of NRF2 protein expression. **a** The protein expression of CERK in mice with acute liver injury was measured via WB at different time points. **b** The mRNA expression of CERK in mice with acute liver injury was measured by qRT‒PCR at different time points. **c** The protein expression of CERK in AML12 cells with oxidative damage induced by different concentrations of H_2_O_2_ was measured via western blotting. **d** The mRNA expression of CERK in AML12 cells with oxidative damage induced by different concentrations of H_2_O_2_ was measured via qRT‒PCR. **e** The protein expression of CERK in AML12 cells with stable CERK knockdown was measured by western blotting. **f** The mRNA expression of CERK in AML12 cells with stable CERK knockdown was measured by qRT‒PCR. **g** The protein expression of CERK in AML12 cells treated with NVP-231 (upper panel) or C1P (lower panel) was measured by western blotting. **h** The mRNA expression of CERK in AML12 cells treated with NVP-231 or C1P was measured by qRT‒PCR. One-way ANOVA was used to analyze the significant differences. ns not significant.

Studies reveal that NRF2 is crucial for oxidative stress^26,27^. Therefore, in this study, the mRNA and protein levels of NRF2 were measured by qRT‒PCR and WB, respectively. In vivo CCl_4_ administration led to an increase in NRF2 protein expression, especially at 48 h, and CERK inhibition by NVP-231 reversed this increase (Fig. 4a). CERK protein expression was increased after CCl_4_ treatment, but NVP-231 did not affect CERK protein expression (Fig. 4a). Interestingly, neither CCl_4_ nor NVP-231 administration affected NRF2 mRNA expression (Fig. 4b). In vitro H_2_O_2_ treatment led to an increase in NRF2 protein expression in a concentration-dependent manner without affecting NRF2 mRNA levels (Fig. 4c, d). The H_2_O_2_-induced increase in NRF2 protein expression could be partly or even completely inhibited by CERK knockdown (Fig. 4e) or NVP-231 treatment (Fig. 4g, upper panel), while NRF2 mRNA expression was not affected (Fig. 4f, h). Moreover, the H_2_O_2_-induced increase in NRF2 protein expression was further enhanced by the addition of C1P in a concentration-dependent manner (Fig. 4g, lower panel); however, NRF2 mRNA expression was not affected (Fig. 4h). These data demonstrated that CERK/C1P affected only NRF2 protein expression but not NRF2 mRNA expression in acute liver injury, thus indicating that CERK/C1P upregulated NRF2 expression via a posttranslational mechanism.

### CERK and C1P facilitate the activation of NRF2 by promoting its dissociation from KEAP1

**Fig. 5:**
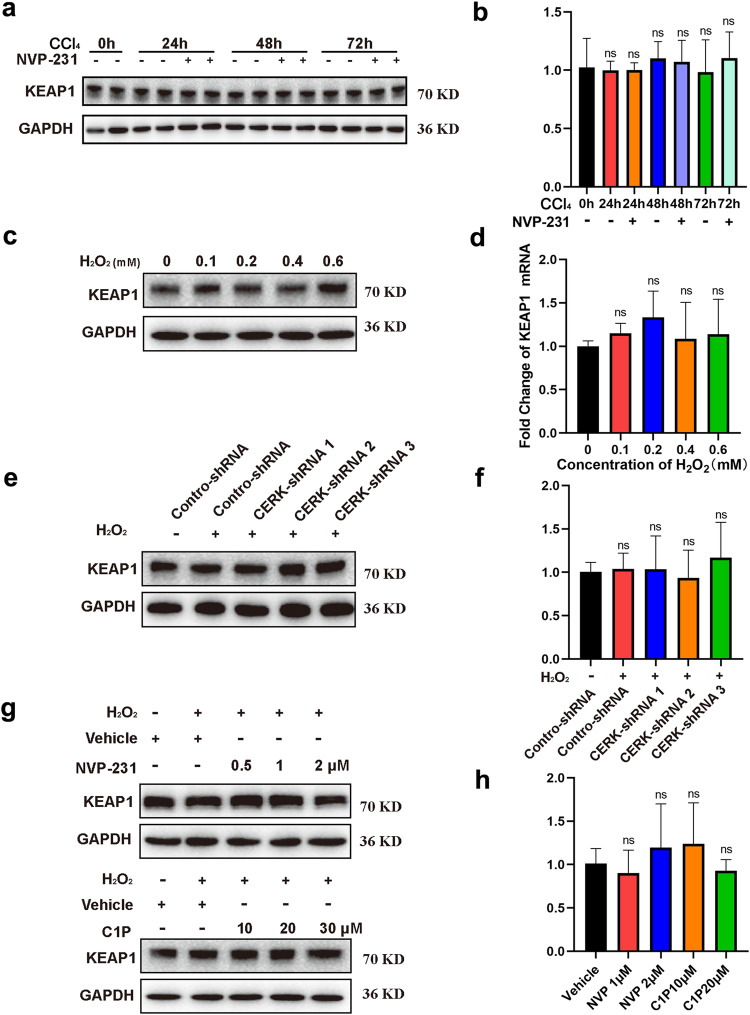
CERK and C1P do not lead to changes in KEAP1 expression, which is not involved in the upregulation of NRF2 protein expression induced by CERK/C1P. **a** The protein expression of KEAP1 in mice with acute liver injury was measured via WB at different time points. **b** The mRNA expression of KEAP1 in mice with acute liver injury was measured by qRT‒PCR at different time points. **c** The protein expression of KEAP1 in AML12 cells with oxidative damage induced by different concentrations of H_2_O_2_ was measured via western blotting. **d** The mRNA expression of KEAP1 in AML12 cells with oxidative damage induced by different concentrations of H_2_O_2_ was measured via qRT‒PCR. **e** The protein expression of KEAP1 in AML12 cells with stable CERK knockdown was measured by western blotting. **f** The mRNA expression of KEAP1 in AML12 cells with stable CERK knockdown was measured by qRT‒PCR. **g** The protein expression of KEAP1 in AML12 cells treated with NVP-231 (upper panel) or C1P (lower panel) was measured via western blotting. **h** The mRNA expression of KEAP1 in AML12 cells treated with NVP-231 or C1P was measured by qRT‒PCR. One-way ANOVA was used to analyze the significant differences. ns not significant.

KEAP1 serves as an inhibitor of NRF2 in the cytosol and facilitates its proteasomal degradation^28,29^. Therefore, we wondered whether KEAP1 was involved in the regulation of NRF2 protein expression by CERK and C1P. Neither CCl_4_ administration nor NVP-231 treatment affected the mRNA or protein level of KEAP1 in vivo (Fig. 5a, b). Similar results were observed when the cells were treated with H_2_O_2_ in vitro (Fig. 5c, d). Moreover, neither CERK inhibition by shRNA or NVP-231 nor C1P supplementation affected the mRNA or protein level of KEAP (Fig. 5e–h). These data suggested that KEAP1 expression was not involved in the CERK/C1P-mediated increase in NRF2 protein levels.

**Fig. 6:**
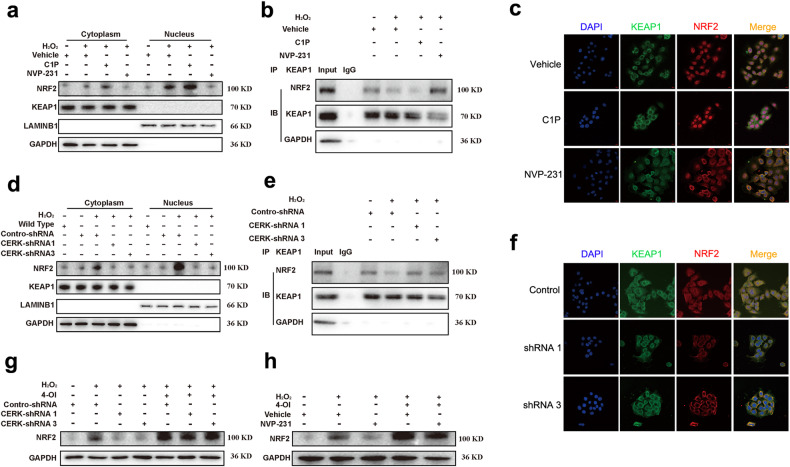
CERK and C1P facilitate the activation of NRF2 by promoting its dissociation from KEAP1. **a** The protein expression of NRF2 and KEAP1 in the cytoplasm and nucleus of AML12 cells treated with C1P or NVP-231 was measured via WB. The negative control groups were not exposed to H_2_O_2_, while the vehicle, C1P, and NVP-231 groups were exposed to H_2_O_2_. **b** The interaction between KEAP1 and NRF2 in AML12 cells treated with C1P or NVP-231 was assessed by co-IP. The negative control groups (lane 2) were incubated with the same quality rabbit IgG as the co-IP baits. **c** Fluorescence images of the colocalization of KEAP1 and NRF2 in AML12 cells treated with C1P or NVP-231 were obtained via IF. **d** The protein expression of NRF2 and KEAP1 in the cytoplasm and nucleus of AML12 cells with stable CERK knockdown was measured via western blotting. **e** The interaction between KEAP1 and NRF2 in stable CERK-knockdown AML12 cells was assessed by co-IP. The negative control groups (lane 2) were incubated with the same quality rabbit IgG as the co-IP baits. **f** IF images of the colocalization of KEAP1 and NRF2 in AML12 cells with stable CERK knockdown were obtained. **g** The protein expression of NRF2 in AML12 cells cotreated with 2 μM NVP-231 and 4-OI was measured by western blotting. **h** The protein expression of NRF2 in stable CERK-knockdown AML12 cells treated with 4-OI was measured by western blotting. One-way ANOVA was used to analyze the significant differences. *P < 0.05, **P < 0.01, ***P < 0.001, ****P < 0.0001, and ns, not significant.

Studies have shown that the E3 ligase adaptor protein KEAP1 associates with NRF2 for its proteasome degradation at steady state^30^. The dissociation of NRF2 from KEAP1 and the subsequent nuclear translocation of NRF2 are the crucial steps in NRF2 activation^31^. Therefore, we explored whether these two steps were impacted by C1P. H_2_O_2_ treatment led to an increase in nuclear NRF2 and a decrease in the interaction between KEAP1 and NRF2; however, this interaction was promoted by C1P supplementation. CERK inhibition by NVP-231 reduced H_2_O_2_-mediated nuclear NRF2 accumulation and promoted the interaction between KEAP1 and NRF2 (Fig. 6a–c). The results for the CERK-knockdown group were similar to those for the NVP-231 group (Fig. 6d–f). However, KEAP1 translocation to the nucleus was not observed, and cytosolic KEAP1 levels were not affected by H_2_O_2_ treatment under the conditions of C1P supplementation or CERK inhibition by NVP-231 or shRNA (Fig. 6a–f). These data suggested that CERK/C1P facilitates the activation of NRF2 by promoting its dissociation from KEAP1. As KEAP1 interacts with NRF2 and subsequently promotes its ubiquitination and degradation, we investigated whether C1P inhibited NRF2 ubiquitination. Indeed, H_2_O_2_ decreased NRF2 ubiquitination in the presence of MG132 (Supplementary Fig. 10a), which was consistent with the increase in NRF2 protein levels observed upon H_2_O_2_ treatment. Moreover, ubiquitination of NRF2 was inhibited by C1P and promoted by CERK inhibition or knockdown (Supplementary Fig. 10b, c).

Then, we performed a rescue experiment using 4-octyl itaconate (4-OI), which promoted the dissociation of NRF2 from KEAP1 and activated NRF2^32^. 4-OI treatment significantly rescued the decrease in cell viability and CAT activity caused by CERK inhibition via NVP-231 (Supplementary Fig. 11a, b). 4-OI treatment also reversed the increase in MDA levels caused by CERK inhibition via NVP-231 (Supplementary Fig. 11c). The decrease in NRF2 protein expression caused by CERK inhibition was reversed using 4-OI (Fig. 6g). Consistently, the results of 4-OI treatment in CERK-knockdown cells were similar to those in the NVP-231 group. (Fig. 6h and Supplementary Fig. 11d–f). These results indicated that CERK/C1P facilitates the activation of NRF2 by promoting its dissociation from KEAP1.

### C1P binds to the DGR domain of KEAP1 to promote the dissociation of NRF2

**Fig. 7:**
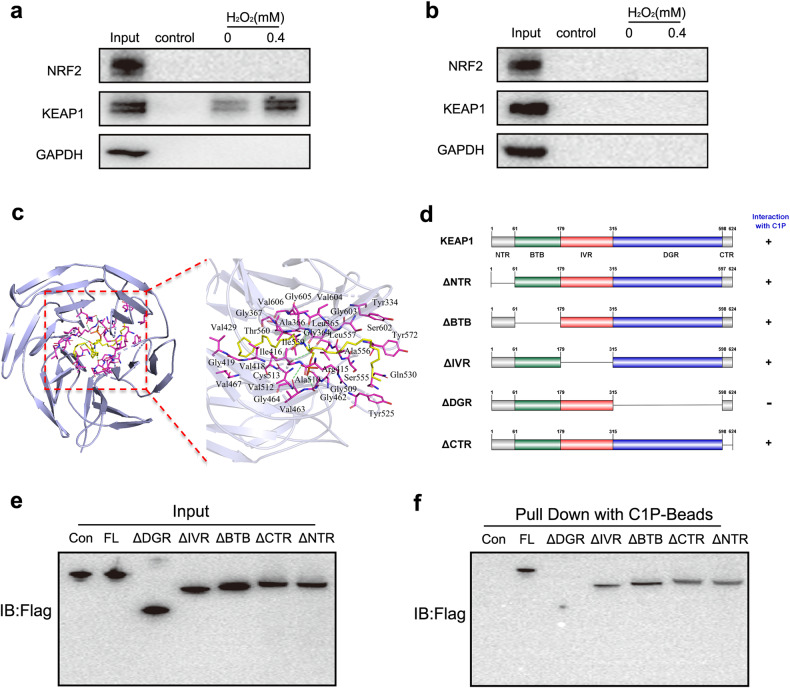
C1P binds to the DGR domain of KEAP1 to promote the dissociation of NRF2. **a** C1P-coated beads were used to pull down the proteins that directly bind to C1P in AML12 cells with H_2_O_2_-induced oxidative damage. The KEAP1 and NRF2 levels in the pulled-down proteins were measured via western blotting. **b** Ceramide-coated beads were used to pull down the proteins that directly bind to ceramide in AML12 cells with H_2_O_2_-induced oxidative damage. The KEAP1 and NRF2 levels in the pulled-down proteins were measured via western blotting. **c** The binding mode and binding site of the C1P and KEAP1 proteins (PDBID: 7C60) were analyzed. AutoDock Vina was used for docking. **d** The domain structure of the mouse KEAP1 protein is shown. Only the deletion of the DGR domain inhibited the binding of C1P to KEAP1. **e** FLAG-tagged full-length KEAP1 and domain deletion mutants of KEAP1 were overexpressed in HEK-293T cells and pulled down with C1P-coated beads. In the pull-down assay of domain deletion mutants of KEAP1 with C1P-coated beads, the input proteins were analyzed. **f** The domain deletion mutants of KEAP1 that can bind to C1P were pulled down and analyzed by western blotting.

Because ceramide and C1P can bind to some proteins directly^33,34^, we hypothesized that C1P might bind to KEAP1 or NRF2 to regulate their interaction. First, we used C1P- and ceramide-coated beads to pull down the proteins that bind to C1P and ceramide, respectively. Interestingly, NRF2 was not detected in either pulldown experiment, while KEAP1 was detected only in the pulldown experiment with C1P-coated beads but not in the experiment with ceramide-coated beads (Fig. 7a, b), which indicated that KEAP1 is the specific binding target of C1P. Furthermore, we conducted a molecular docking simulation to investigate the binding between C1P and KEAP1 (PDBID:7C60). Multiple hydrogen bond interactions formed between C1P and the amino acid residues of KEAP1, including Gly462, Val463, Ala510, Ile416, Gly364, and Val604. Furthermore, hydrophobic interactions formed between C1P and some amino acid residues of KEAP1, including Tyr334, Ser602, Tyr572, Gln530, Ser555, Tyr525, Gly509, Val463, Arg415, Ala556, Leu557, Leu365, Ile559, Gly464, Val512, Cys513, Val418, Val467, Gly419, Val429, Thr560, Ala366, Gly367, Val606, Gly605, and Gly603 (Fig. 7c). We found that almost all the predicted binding sites between C1P and KEAP1 were present in the double glycine repeat (DGR) domain, which is the NRF2 binding site (Fig. 7d). Then, we constructed plasmids harboring and sequences with deletion mutants in each KEAP1 domain, namely, Flag-KEAP1-dNTR, Flag-KEAP1-dBTB, Flag-KEAP1-dIVR, Flag-KEAP1-dDGR, and Flag-KEAP1-dCTR, and transfected HEK-293T cells to express the mutant proteins for pulldown experiments (Fig. 7d). C1P was incapable of binding to KEAP1 only when the DGR domain was deleted (Fig. 7e, f). These results suggested that C1P binds to the DGR domain of KEAP1 to induce the dissociation of NRF2 from KEAP1.

### CERK is associated with the severity of human liver injury

**Fig. 8:**
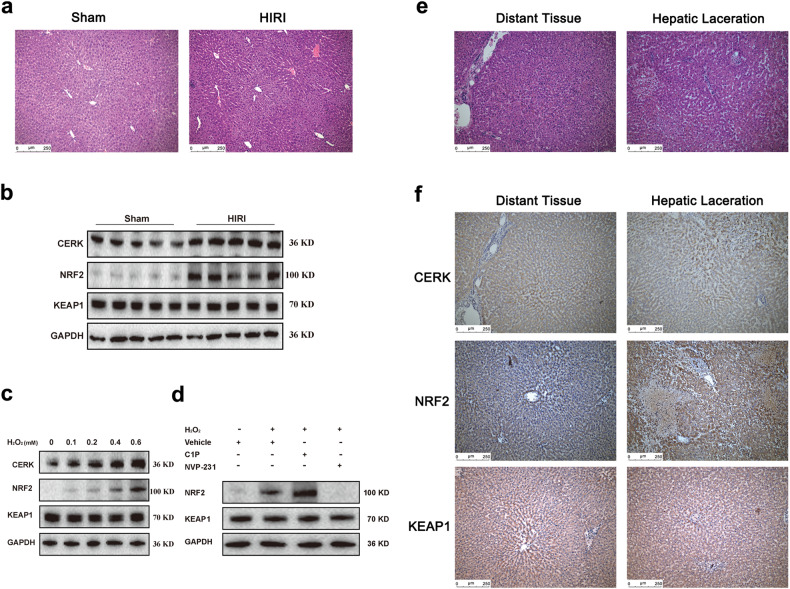
CERK is associated with the severity of liver injury in humans. **a** Representative images of mice subjected to HIRI and stained with hematoxylin and eosin (H&E) are shown. Scale bar: 250 μm. **b** The protein expression levels of CERK, NRF2, and KEAP1 in mice with HIRI were determined (*n* = 5/group). **c** The protein expression levels of CERK, NRF2, and KEAP1 in human primary hepatocytes treated with different concentrations of H_2_O_2_ for 24 h were determined (*n* = 3/group). **d** The protein expressions of NRF2 and KEAP1 were determined in human primary hepatocytes treated with 20 μM C1P or 2 μM NVP-231 for 24 h and exposed to H_2_O_2_ (0.4 mM) (*n* = 3/group). **e** Representative images of H&E staining from patients with hepatic lacerations are shown. Scale bar: 250 μm. **f** Representative images of IHC staining for CERK, NRF2, and KEAP1 in patients with hepatic lacerations are shown. Scale bar: 250 μm.

The aforementioned effects of CERK and C1P were confirmed in hepatic ischemia‒reperfusion injury (HIRI), which is a well-characterized model of acute liver injury and a clinically significant process associated with severe impairment of energy metabolism and oxidative stress^35^. The expression levels of CERK, NRF2, and KEAP1 were detected in the livers of mice subjected to HIRI. H&E staining revealed that laparotomy did not result in liver injury in the sham group, while some pathological changes, such as necrosis, the destruction of lobules, and inflammatory cell infiltration, were observed in the HIRI group (Fig. 8a). ALT, AST, and LDH levels were significantly increased in the HIRI group compared to that in the sham group (Supplementary Fig. 12a–c). The expression of CERK and NRF2 was significantly upregulated in the HIRI group, while no significant change in KEAP1 was observed (Fig. 8b).

Our findings in mice were validated in human primary hepatocytes exposed to H_2_O_2_ and patients with a hepatic laceration after trauma. First, we determined the expression of CERK, KEAP1, and NRF2 in human primary hepatocytes exposed to H_2_O_2_. As expected, with increasing H_2_O_2_ concentration, the viability of human primary hepatocytes decreased in a dose-dependent manner (Supplementary Fig. 13a). CERK and NRF2 expression was gradually upregulated, while no significant change in KEAP1 was observed (Fig. 8c). In human primary hepatocytes exposed to 0.4 μM H_2_O_2_, NVP-231 treatment decreased cell viability, while C1P treatment increased cell viability (Supplementary Fig. 13b). NRF2 protein expression was downregulated by NVP-231 treatment and upregulated by C1P addition, while no significant change in KEAP1 was observed (Fig. 8d). Similar results were observed in patients with hepatic lacerations. In contrast to that in the distant tissue 2 cm from the edge of the laceration, the dilated hepatic sinusoid, inflammatory cell infiltration, and focal areas of necrosis appeared in the hepatic laceration group (Fig. 8e). The expressions of CERK and NRF2 were upregulated in the hepatic laceration group, while the expression of KEAP1 was not significantly different (Fig. 8f).

## Discussion

**Fig. 9:**
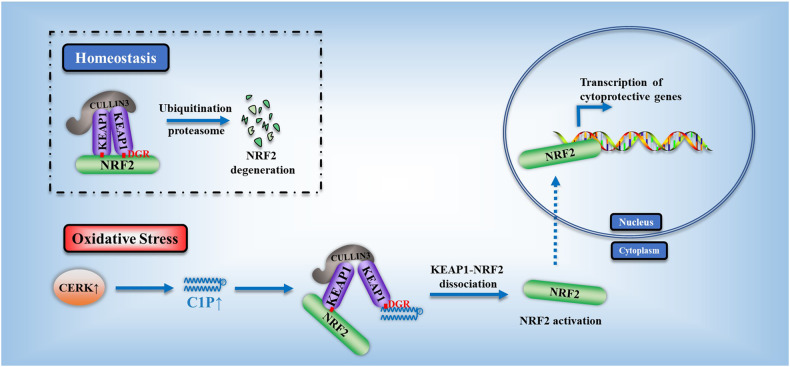
The schematic depiction shows the mechanism underlying the regulatory effects of CERK-mediated C1P on NRF2 through binding to KEAP1. Oxidative stress in the liver induces upregulated CERK expression, and CERK mediates the accumulation of C1P, which binds to the DGR region of KEAP1 and disrupts the binding of KEAP1 to NRF2. Then, NRF2 escapes KEAP1-mediated degradation and translocates to the nucleus, where it promotes the transcription of antioxidant genes.

Herein, we demonstrated that C1P generated by CERK binds to KEAP1 via the DGR domain, leading to KEAP1-NRF2 dissociation and thereby promoting the translocation of NRF2 to the nucleus, where NRF2 exerts antioxidant effects. Our findings revealed that CERK and C1P play a crucial role in regulating the antioxidant response; furthermore, we elucidated the underlying mechanism by which these molecules regulate redox homeostasis (Fig. 9).

Because SPL metabolic dysregulation is involved in liver injury^36,37^, we identified dysregulated genes in the SPL metabolism pathway in liver tissue from mice that underwent acute CCl_4_ toxicity. Through screening and validation, we found that CERK was the SPL gene with the most prominently upregulated expression, indicating that CERK might be involved in acute liver injury. CERK is the only enzyme that can generate C1P, and C1P levels are substantially increased in some kinds of damaged tissues; however, the functional roles of CERK and C1P in tissue injury remain controversial. On the one hand, C1P was initially described as a proinflammatory factor that acts through the activation of cPLA2α and was recently demonstrated to induce autophagy and inflammation^38^, which suggested that C1P might aggravate tissue injury by promoting inflammation. On the other hand, some studies have indicated that C1P has an anti-inflammatory function^39,40^ and can protect against tissue injury^41,42^. Therefore, the role of C1P in inflammation remains controversial. In this study, we demonstrated that C1P protected against acute liver injury and confirmed that its protective effect was mediated by its antioxidant effect. However, C1P may also alleviate acute liver injury by reducing inflammation. Our findings also revealed that SPHK1 and SGPP2, the main enzymes that generated and degraded S1P, were both highly expressed during acute liver injury. Similarly, numerous studies have demonstrated that S1P plays a role in the regeneration and inflammation associated with various liver injuries, such as hepatic ischemia‒reperfusion^43^, liver fibrosis^44^, and hepatitis^45^. For instance, in a study on carbon tetrachloride-induced acute liver injury, SPHK1 mRNA expression and activity increased at 72 h, and S1P facilitated hepatic wound healing through S1PR2^46^, which suggested that S1P plays a role in liver regeneration following acute injury. Additionally, S1P protects against cell death^47,48^. Thus, we speculate that S1P may also participate in acute liver injury.

Regarding the function of CERK or C1P in regulating redox homeostasis, earlier studies showed that C1P moderately increased ROS production to exert a mitogenic effect via NADPH oxidase activation^49^. In contrast, several studies have shown that CERK inhibition increases mitochondrial ROS production in Arabidopsis^50–52^. Importantly, recent research revealed that inhibition of CERK increases VDAC-mediated mitochondrial membrane potential and generates cellular ROS in non-small cell lung cancer with KRAS mutations^53^. However, the mechanism underlying the role of CERK or C1P in redox homeostasis is poorly understood. We found that CERK-mediated C1P generation upregulated the protein expression of NRF2. Although several studies have reported that SPL metabolism has a strong but complex association with the NRF2 signaling pathway^18–23^, little is known about the relationship among CERK, its metabolite C1P, and the NRF2 signaling pathway. Furthermore, we demonstrated that CERK/C1P regulates NRF2 at the protein level but not at the transcription level, which suggested that C1P might be involved in posttranslational modification of NRF2. KEAP1, which is a well-established repressor of NRF2, mainly affects NRF2 posttranslational modification. Therefore, we hypothesized that C1P promotes the NRF2 signaling pathway by regulating KEAP1. Although our results showed that C1P did not affect KEAP1 protein levels, it disrupted the association between NRF2 and KEAP1, inhibited NFR2 degradation, and promoted the nuclear translocation of NFR2. Similarly, previous studies have shown that p62/SQSTM1 and gankyrin can bind directly to KEAP1 and prevent NRF2 degradation without regulating KEAP1 expression^54,55^. Moreover, several regulators, such as p21^56^ and esculetin^57^, have been shown to influence the interaction between NRF2 and KEAP1 by competing with NRF2 for binding to KEAP1. Interestingly, Scott et al.^58^ reported that C1P could bind to HSD17B4, which is a multifunctional enzyme that is important for fatty acid oxidation and related to the activity of peroxisomes and mitochondria^58,59^. These findings suggested that C1P might regulate oxidative activity by binding directly to proteins involved in redox homeostasis. Consistent with these findings, our work revealed that C1P could bind to KEAP1 but not to NRF2. The detailed binding site of C1P was located in the DGR domain, which also binds NRF2 directly, indicating that C1P competes with NRF2 for binding to KEAP1.

Elevated CERK expression might constitute a defense mechanism by which the liver responds to oxidative damage, and CERK expression returns to normal with the restoration of oxidative stress. Our results may also support the findings of a previous study that showed the close relationship between CERK and vitamin E in NAFLD model mice^17^. Vitamin E, which is a well-established antioxidant, prevents hepatic oxidative stress in NAFLD model mice and restores the level of CERK, which might be a defense mechanism against oxidative stress. In addition, liver injury and inflammation are often related to the initiation of and predisposition to liver carcinogenesis. Accumulating research has shown that CERK and its metabolite C1P play complex regulatory roles in several kinds of tumors, such as leukemia, breast cancer, lung cancer, neuroblastoma, pancreatic cancer, and prostate cancer. Hsieh SY et al. reported that when a hormetic response was induced in human hepatoma cells, CERK expression was upregulated, and silencing CERK increased therapeutic efficacy^60^; however, the exact mechanism underlying the regulation of CERK remains to be elucidated. The activation of NRF2 is crucial for modulating tumor metabolism to suppress various forms of stress and enhance drug tolerance^61,62^; our findings showed a close relationship between NRF2 and CERK, and this finding could provide a new understanding of the role of CERK in tumor progression and resistance to therapy.

KEAP1 interacts with the DLG and ETGE motifs of NRF2 in a two-site binding model The two motifs with different binding affinities to KEAP1 appear to function as hinges and latches for the interaction between NRF2 and KEAP1^63^. The high-affinity ETGE motif allows stable binding of NRF2 to KEAP1 for KEAP1-mediated degradation of NRF2 by acting as a hinge with KEAP1. The weak-affinity DLG motif is vulnerable and sensitive to modification by the KEAP1-NRF2 complex in the oxidative stress response and is crucial for the activation of NRF2, acting as a latch to lock and unlock the stress response. The hinge-and-latch mechanism provides a sensitive and rapid response to oxidative stress. The DLG peptide has intermolecular electrostatic interactions with amino acids in the DGR and CTR domains of KEAP1, including Asn-382, Ser-508, Arg-415, Arg-483, Ser555 Ser602, and Gly-603^64^. Most of these residues overlapped with our predicted binding sites of C1P to KEAP1, suggesting that C1P might occupy the binding sites between the DLG motif of NRF2 and KEAP1 to disrupt NRF2-KEAP1 complex formation.

In conclusion, we demonstrated that CERK expression is upregulated in acute liver injury and that elevated levels of CERK and C1P are associated with antioxidant activity. CERK/C1P plays an antioxidant role by activating NRF2 signaling. Further mechanistic studies revealed that C1P, which is generated by CERK, can lead to the dissociation of KEAP1 from NRF2 by competing with NRF2 for binding to the DGR domain of KEAP1. Thus, C1P facilitates the nuclear translocation of NRF2. The regulation of CERK and C1P might be a novel therapeutic strategy for acute liver injury, chronic liver disease, and even hepatocellular carcinoma.

### Supplementary information

Supplementary Information
