# Supplementary material for: Ceramide kinase-mediated C1P metabolism attenuates acute liver injury by inhibiting the interaction between KEAP1 and NRF2
Source: Exp Mol Med. 2024 Apr 1;56(4):946–58. doi: 10.1038/s12276-024-01203-4 (PMC11059394; doi:10.1038/s12276-024-01203-4)
Supplement: Supplementary file 1 — Supplementary Information [file 12276_2024_1203_MOESM1_ESM.pdf]

Supplementary Information

Supplementary materials and methods

Cell lines

Normal mouse hepatocytes cell line AML12 were purchased from Cellcook (Guangzhou, China). LO2 and HEK293T were gifts from professor Juan Wang's lab. AML12 was cultured in DMEM-F12 (GIBCO, USA) supplemented with 10% fetal bovine serum (FBS), 40 ng/mL dexamethasone, ITS liquid media supplement, penicillin and streptomycin. LO2 and HEK293T were maintained in DMEM (GIBCO, USA) supplemented with 10% fetal bovine serum (FBS), penicillin and streptomycin.

Animal experiments

C57BL/6J mice were bought from Hunan Slyke Jingda Laboratory Animal Co. LTD (Hunan, China). All mice were housed in specific pathogen-free (SPF) conditions under a 12h light-dark diurnal cycle with controlled temperature (20-26℃).

All animal experiments were conducted in accordance with the Guide for the Care and Use of Laboratory Animals (NIH publications no. 80-23, revised 1996) and approved by the institutional ethical guidelines of Guilin medical college.

Mice were randomly divided into each group during each animal experiment. For in vivo experiments of CCl_4 concentrations, Male C57BL/6J mice were injected with different doses of CCl_4 or vehicle (Olive oil). For In vivo experiments of NVP-231, all mice received a single CCl_4 injection (1 ml/kg). Simultaneously, mice received a daily NVP-231 injection (0.4 mg/kg). All injections were administered intraperitoneally (i.p).

Clinical Specimens

Human liver tissues of hepatic laceration (n=5) were obtained from the Affiliated Hospital of Guilin Medical University (Guilin, China). The patients with a hepatic laceration underwent liver segment resection. Clinical samples were collected from patients after obtaining informed consent in accordance with a protocol approved by the Ethics Committee of the Affiliated Hospital of Guilin Medical University (Guilin, China).

Reagents

The primary antibodies used for western blotting included: CERK (25731-1-AP, Proteintech, China), GAPDH (AF0006, Beyotime, China), NRF2 (16396-1-AP, Proteintech, China), KEAP1(10503-2-AP, Proteintech, China), LAMIN B1 (ab133741, Abcam, US), FLAG (M20008M, Abmart, China), and ubiquitin (80992-1-RR, Proteintech, China).

In the co-immunoprecipitation, we used the rabbit-derived anti-KEAP1 (10503-2-AP, Proteintech, China) and the mouse-derived anti-NRF2 (66504-1-Ig, Proteintech, China) antibody as baits and normal rabbit IgG antibody (A7016, Beyotime, China) and normal mouse IgG antibody (A7028, Beyotime, China) as the negative control. To avoid the confounding effects of co-immunoprecipitation(co-IP) baits, we used the primary antibodies from different species.

The primary antibodies used for IHC and immunofluorescence (IF) included: CERK (25731-1-AP, Proteintech, China), NRF2 (16396-1-AP, Proteintech, China), rabbit-derived KEAP1(10503-2-AP, Proteintech, China), mouse-derived KEAP1(60027-1-Ig, Proteintech, China).

Other reagents included: Custom RT2 Profiler PCR array for sphingolipid pathway (Qiagen, USA); Mouse Ceramide-1-phosphate (C1P) ELISA Kit (MyBioSource, USA); Ceramide-1-phosphate (d18:1/2:0) (Avanti polar lipids, USA); Ceramide coated beads and C1P coated beads (Echelon,

USA); 4-Octyl Itaconate (OI) and NVP-231 (MedChemExpress, USA); MDA and CAT assay kits (Solarbio, China); ROS assay kit(Beyotime, China); ALT, AST, and LDH Assay Kits (Roche, USA).

Quantitative real-time PCR (qRT–PCR)

Total RNA was extracted from liver tissues or cell lines with TRIzol (TAKARA, Japan). Then, the RNA was reverse-transcribed to cDNA. qRT–PCR was performed according to the manufacturer's instructions on a 7500 Real-Time PCR System (Thermo Fisher, MA, USA) using TB Green Premix Ex Taq II (TaKaRa, Dalian, China). The relative expression of the target genes was normalized to that of the control, GAPDH, using the $2^{-\Delta\Delta Ct}$ method.

Western blotting

Total protein was extracted from liver tissues or cells by RIPA buffer (Solarbio, China) containing a protease inhibitor. The cytoplasmic and nuclear proteins were extracted following the instructions of the cytoplasmic and nuclear fractionation kit for cells (SC-003, Beijing, China) or for frozen/fresh tissues (NT-032, Invent, China). The extracted proteins were boiled, loaded onto, separated with 10% sodium dodecyl sulfate-polyacrylamide (SDS–PAGE) gels, and transferred to polyvinylidene difluoride membranes. The membranes were subsequently blocked in TBS-Tween with 5% skim milk for 1 h at room temperature. Then, the membranes were incubated overnight at 4°C with primary antibodies. After washing, the membranes were incubated for 1 h at room temperature with an HRP-conjugated secondary antibody (Ray, China) and then incubated with a Super ECL detection reagent (Yeasen, China). The immunoreactive bands were visualized by a Tanon Automatic Chemiluminescence and Fluorescence Image Analysis System (Tanon, China).

Immunohistochemistry (IHC)

After drying, dewaxing, and rehydration, paraffin-embedded liver tissue sections were subjected to

high-pressure antigen repair with citrate buffer (0.01 M, pH 6.0) and were subsequently incubated in 0.3% H_2O_2 solution for 10 min to block endogenous peroxidase activity. Then, the sections were blocked in PBS-Tween with 5% BSA for 1 h and incubated with rabbit polyclonal anti-CerK antibody (1:200) at 4°C overnight. Then, the sections were incubated with a second antibody for 1 h and stained with 3,3'-diaminobenzidine tetrahydrochloride (DAB) and then hematoxylin. Then, the sections were dehydrated, cleared, sealed with coverslips, and analyzed by microscopy.

Immunofluorescence

A total of 4×10^4 cells were plated in 24-well plates for 24 h. After treatment, the cells were fixed with 4% PFA for 10 min, permeabilized with 0.1% Triton X-100 for 10 min, incubated with a rabbit anti-NRF2 antibody and mouse anti-KEAP1 antibody, incubated with Alexa-conjugated secondary antibodies, and finally stained with DAPI. Images were acquired by a Nikon AXR confocal microscope.

Enzyme-linked immunosorbent assay (ELISA)

The C1P levels in tissues or cell lysates were quantified using enzyme-linked immunosorbent assay (ELISA) kits according to the manufacturer's protocol.

Cell treatments

For C1P treatment, cells were treated with 0.4 mM H_2O_2 and different concentrations of C1P for 24 h. For NVP-231 treatment, cells were treated with 0.4 mM H_2O_2 and different concentrations of NVP-231 for 24 h. For C1P supplementation assays in shRNA-CERK cells, control and shCERK cells were treated with 0.4 mM H_2O_2 and 20 μM C1P for 24 h. For 4-Octyl Itaconate (OI) treatment, cells were treated with 0.4 mM H_2O_2 and C1P or NVP-231 for 24 h. After 12 h, cells were treated with 62.5 μM 4-OI for 12 h.

Vector construction

To generate pLV-shCERK and pLV-shNC, 3 short hairpin RNAs targeting mouse CERK (shCERK) or non-target shRNA (shNC) were cloned to the lentiviral vector pLV-U6. To generate pLV-KEAP1, genes of full-length mouse KEAP1 and KEAP1 with different domains deletion were cloned into the lentiviral vector pLV-EF1A. All KEAP1 genes were tagged by FLAG. Sequences of shRNAs and different KEAP1 domains are listed in Supplementary Table 1.

Gene expression and RNA interference

HEK293T cells were used to produce lentiviruses. For each dish, 4 μg of pLV-Flag, pLV-Flag-KEAP1-FL, pLV-Flag-KEAP1- dNTR, pLV-Flag-KEAP1-dBTB, pLV-Flag-KEAP1-dIVR, pLV-Flag-KEAP1-dDGR, pLV-Flag-KEAP1-dCTR, pLV-shNC or pLV-shCERK plasmid and packaging plasmids (3 μg psPAX and 1 μg pMD2.G) were cotransfected into HEK293T cells using Lipofectamine 3000. After 48 and 72 hours, viruses were harvested and concentrated using a lentivirus concentration kit (Genomeditech, China). AML12 cells were infected with this collected lentivirus. After AML12 cell infection with lentivirus, puromycin was used to select a stable cell line.

Data availability

The data in this study are available within the article and the supplementary files.

Supplementary Table 1

REAGENT or RESOURCE	SOURCE	IDENTIFIER
**Oligonucleotides**		
CERK shRNA-1: GGATCTCCACGGGACAATAAACTC GAGTTTATTGTCCCGTGGAGATCC	This paper	N/A
CERK shRNA-2: TCCAGTGGCCGATGGCATAAACTC GAGTTTATGCCATCGGCCACTGGA	This paper	N/A
CERK shRNA-3: CATCGGCTTTGCACATCATTACTCG AGTAATGATGTGCAAAGCCGATG	This paper	N/A
Mus-CERK qPCR forward primer: CGGTACTGGTGTCGGAGATCA	This paper	N/A
Mus-CERK qPCR reverse primer: GTGAATGCGAACGGATTTTCC	This paper	N/A
Mus-GAPDH qPCR forward primer: AACTTTGGCATTGTGGAAGG	This paper	N/A
Mus-GAPDH qPCR reverse primer: CACATTGGGGGTAGGAACAC	This paper	N/A
Mus-KEAP1 qPCR forward primer: TGCCCCTGTGGTCAAAGTG	This paper	N/A
Mus-KEAP1 qPCR reverse primer: GGTTCGGTTACCGTCCTGC	This paper	N/A
Mus-NRF2 qPCR forward primer: TCTTGGAGTAAGTCGAGAAGTGT	This paper	N/A
Mus-NRF2 qPCR reverse primer: GTTGAAACTGAGCGAAAAAGGC	This paper	N/A
Mus-SPHK1 qPCR forward primer: AAAATACTGAGAAACTCGGTCGG	This paper	N/A
Mus-SPHK1 qPCR reverse primer: GCATCGCTTCTTAAAGTCCAGA	This paper	N/A
Mus-SMPDL3B qPCR forward primer: CAGGGGCTCAACTAGGGAG	This paper	N/A
Mus-SMPDL3B qPCR reverse primer: GGGCCAGCATTTAGCACAG	This paper	N/A
Mus-B4GALT6 qPCR forward primer: GGGTCTCCAATCGCTCTCTG	This paper	N/A
Mus-B4galt6 qPCR reverse primer: ATAAAGAGGTACGTGTTGGCG	This paper	N/A
Mus-SMPD3 qPCR forward primer: ACACGACCCCCTTTCCTAATA	This paper	N/A
Mus-SMPD3 qPCR reverse primer:	This paper	N/A

GGCGCTTCTCATAGGTGGTG		
Mus-SGPP2 qPCR forward primer: TTCACCCACTGGAATATCGACC	This paper	N/A
Mus-SGPP2 qPCR reverse primer: AAGTCTCACAACGGGAGGAAA	This paper	N/A
Mus- CerS4 qPCR forward primer: CTGGTGGCTGTGCGAATTG	This paper	N/A
Mus- CerS4 qPCR reverse primer: CCGGGTTGGGCTTTATCTTTC	This paper	N/A

The gene sequence of KEAP1 was obtained from Kit I. Tong's research.[1]

REFERENCES

1.Tong, K.I, et al. Keap1 recruits Neh2 through binding to ETGE and DLG motifs: characterization of the two-site molecular recognition model. *Mol. Cell. Biol.* **26**, 2887-2900 (2006)

Supplementary Figures and Figure legends

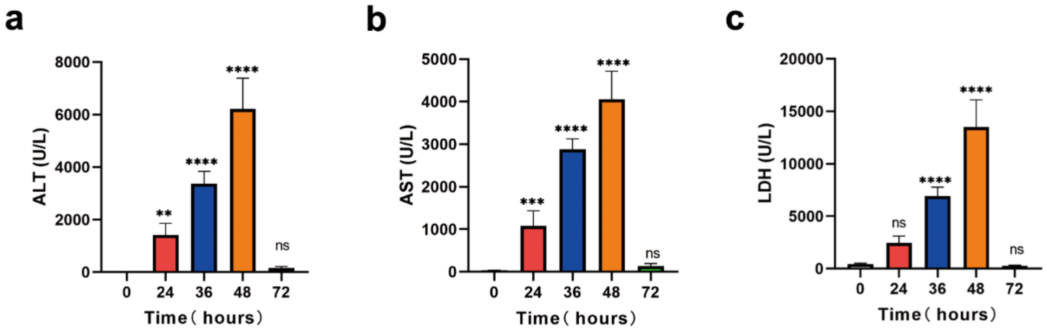

Supplementary Fig. 1. The liver enzymes in time-course experiments of acute liver injury. The serum levels of ALT (a), AST (b), and LDH (c) in time-course experiments of acute liver injury. The number of mice was 5 in each group at each time point. One-way ANOVA was used to analyze the significant differences. ** $p < 0.01$, *** $p < 0.001$, **** $p < 0.0001$.

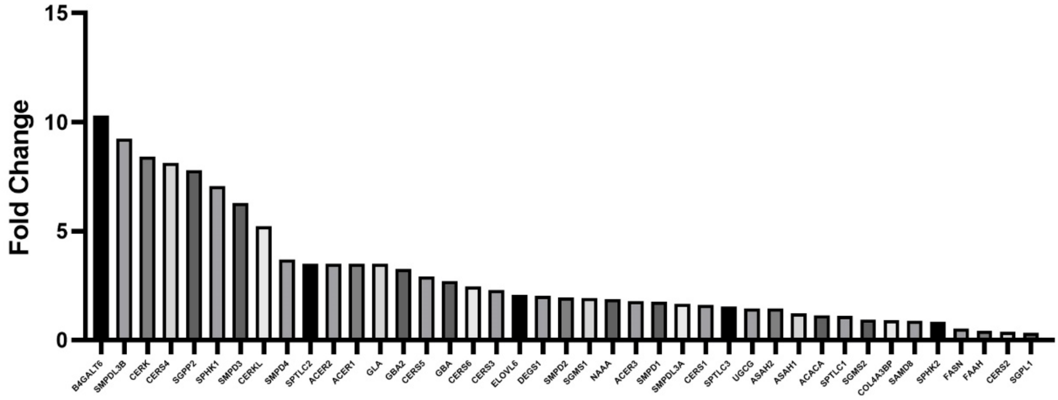

Supplementary Fig. 2. The results of the sphingolipid metabolism PCR array in mice with acute liver injury (n=1) and normal mice (n=1).

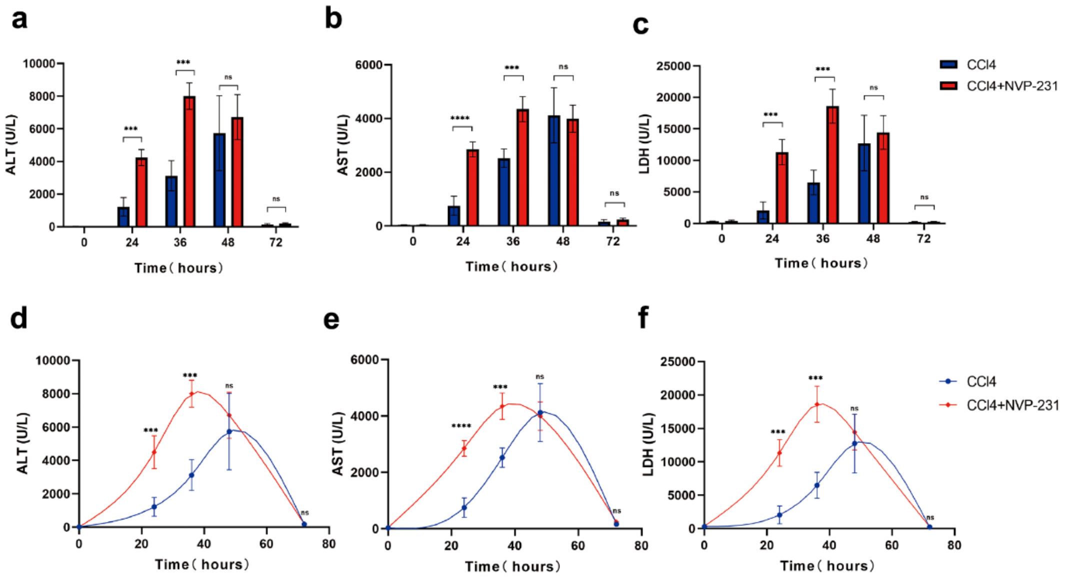

Supplementary Fig. 3. The liver enzymes in time-course experiments of acute liver injury with NVP-231.

(a-c). The serum levels of ALT (a), AST (b), and LDH (c) in time-course experiments of acute liver injury with CERK inhibition. (n=5/group)

(d-f). The continuous curves of ALT (d), AST (e), and LDH (f) in time-course experiments of acute liver injury with CERK inhibition reflected gradual variation of these indicators. (n=5/group)

two-way ANOVA was used to analyze the significant differences. ** $p < 0.01$, *** $p < 0.001$, **** $p < 0.0001$

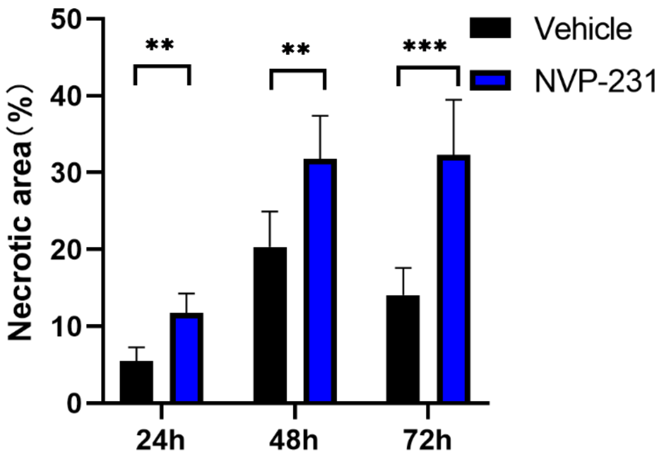

Supplementary Fig. 4. The quantitative analysis of the necrosis area in the NVP-231 group. (n=5/group)

Student t-test was used to analyze the significant differences. ** $p < 0.01$, *** $p < 0.001$

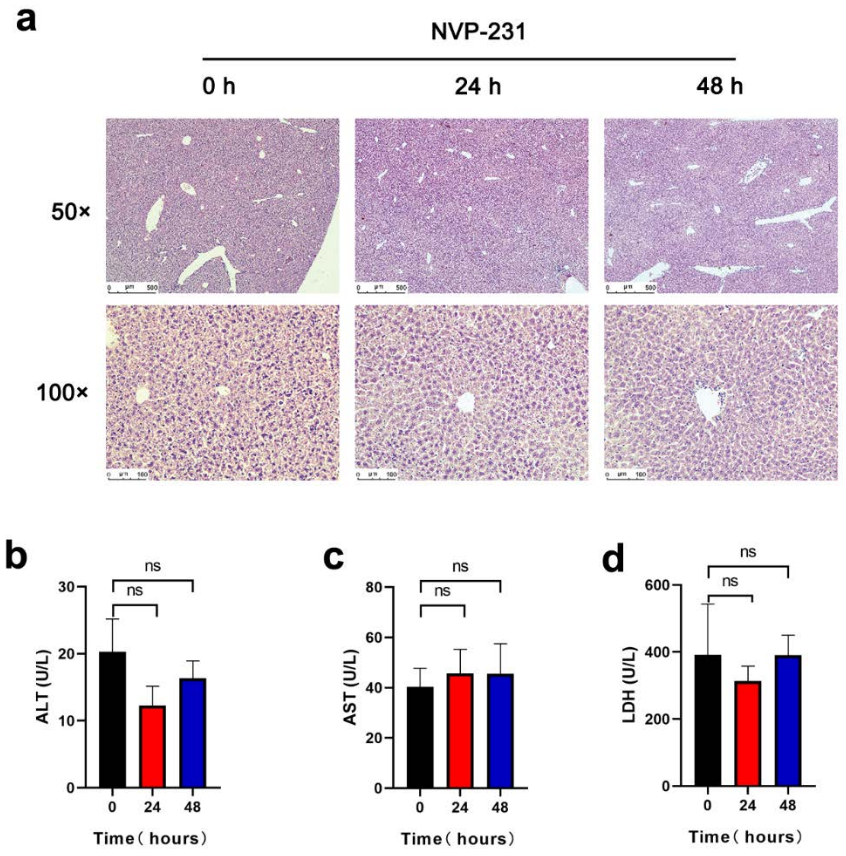

Supplementary Fig. 5. Treatment with NVP-231 alone causes almost no hepatotoxicity.

(a). Representative images of liver injury stained with hematoxylin and eosin (H&E) are shown. Mice in the 24 h and 48 h groups were injected daily with NVP-231 and were harvested at 24 h after the last injection, respectively. The results were compared to those of the 0 h group without any injection. Scale bar: 500 μm (top), 250 μm (bottom).

(b). The serum levels of ALT (b), AST (c), and LDH (d) in mice treated with NVP-231 alone. The number of mice was 3 in each group for each time point.

One-way ANOVA was used to analyze the significant difference. ns indicates no significant difference.

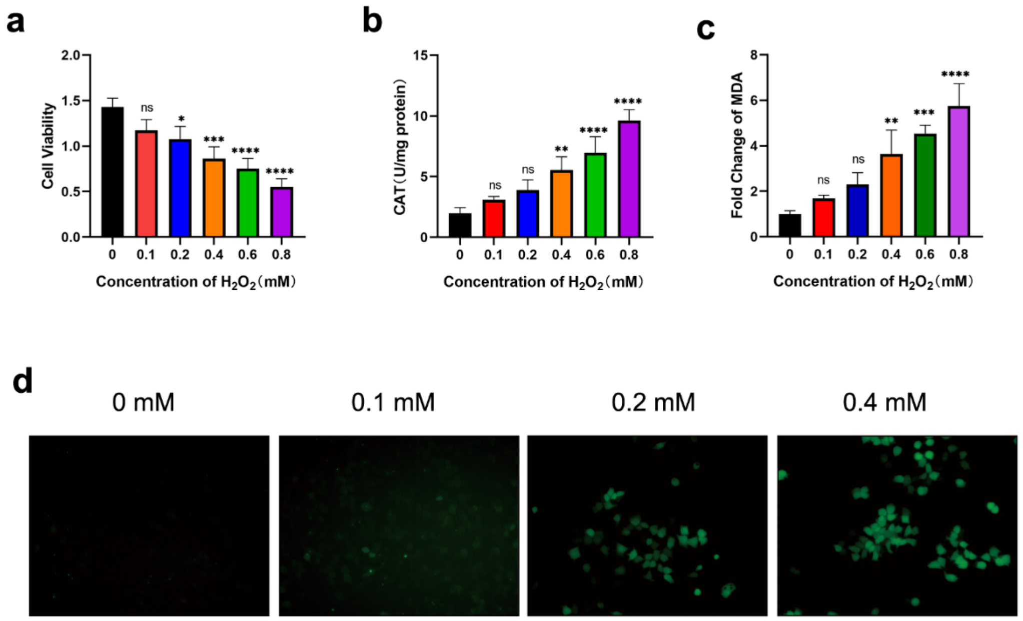

Supplementary Fig. 6. H_2O_2-induced oxidative damage was aggravated in a dose-dependent manner.

(a). The viability of AML12 cells was treated with different concentrations of H_2O_2 for 24 h to induce oxidative damage. (n=3/group)

(b and c). The CAT activity (b) and MDA (c) level of groups treated with H_2O_2 were determined by a CAT Activity and MDA Assay Kit. (n=3/group)

(d). H_2O_2-induced oxidative damage was analyzed by an ROS Assay Kit. (n=3/group)

(a-c) one-way ANOVA. * $p < 0.05$,** $p < 0.01$, *** $p < 0.001$, **** $p < 0.0001$, ns, no significant.

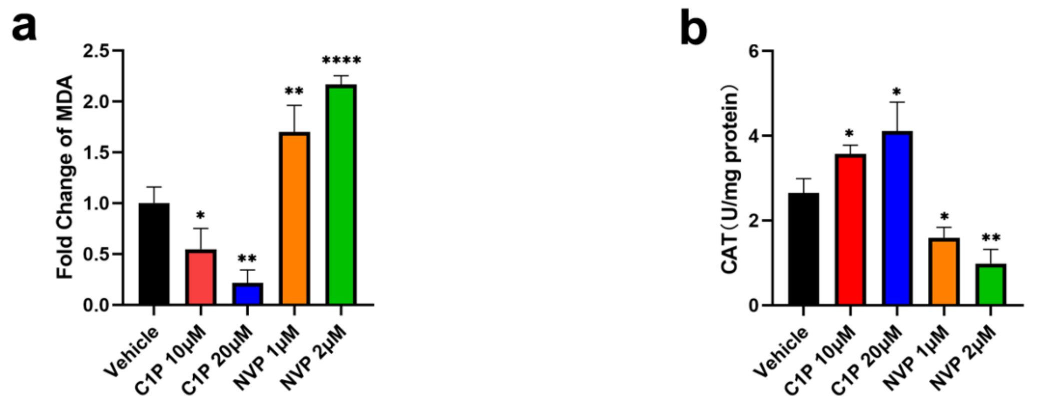

Supplementary Fig. 7. The MDA level (a) and CAT activity (b) of groups treated with C1P and NVP-231 were measured by CAT Activity and MDA Assay Kit. (n=3/group)

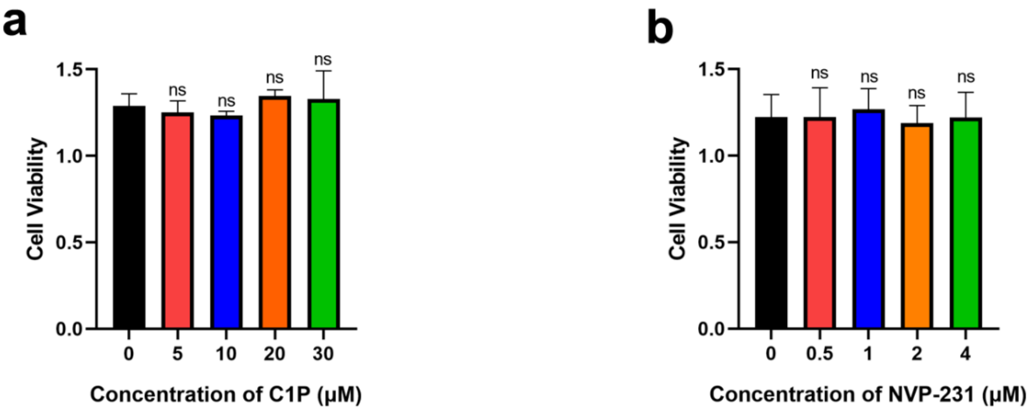

Supplementary Fig. 8. The effects of treatment with C1P or NVP-231 alone in AML12 cells.

(a). The viability of AML12 cells treated with different concentrations of C1P without H_2O_2 treatment. (n=3/group)

(b). The viability of AML12 cells treated with different concentrations of NVP-231 without H_2O_2 treatment. (n=3/group)

One-way ANOVA was used to analyze the significant difference. ns indicates no significant difference.

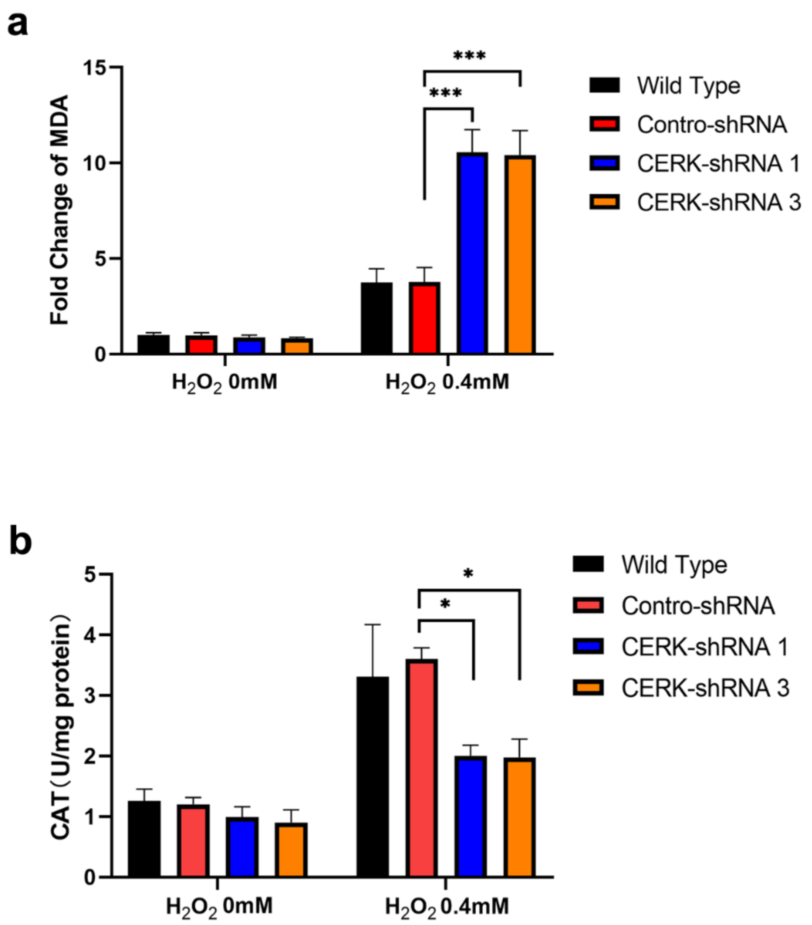

Supplementary Fig. 9.

The MDA level (a) and CAT activity (b) in AML12 cells with stable CERK-knockdown were measured by Kits. (n=3/group)

one-way ANOVA.* $p < 0.05$, *** $p < 0.001$.

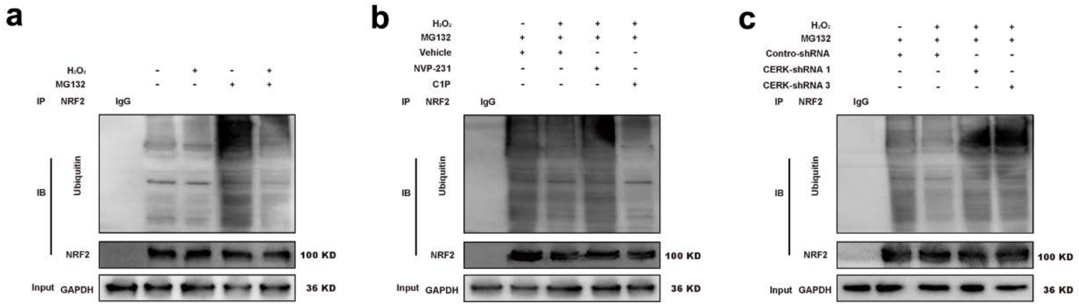

Supplementary Fig. 10. The effects of C1P or CERK on ubiquitination of NRF2.

(a). AML12 cells were treated with 0.4 mM H_2O_2 for 24 h or 10 μM MG132 for 12 h.

(b). AML12 cells were treated with 20 μM C1P or 2 μM NVP-231 for 24 h when exposed to 0.4 mM H_2O_2 and 10 μM MG132.

(c). CERK-knockdown AML12 cells were treated with 0.4 mM H_2O_2 for 24 h or 10 μM MG132 for 12 h.

Immunoprecipitated using anti-NRF2 antibody. NRF2 ubiquitination and GAPDH proteins were detected by western blotting.

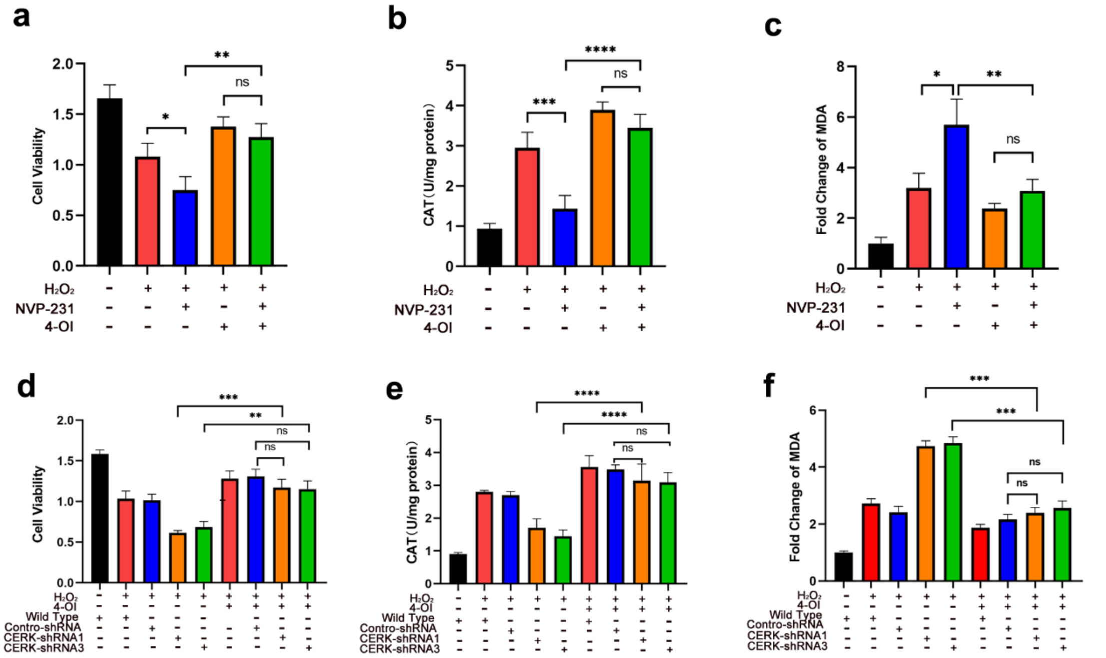

Supplementary Fig. 11. 4-OI treatment rescued the hepatocyte oxidative damage caused by CERK inhibition.

(a). The viability of AML12 cells cotreated with 2 μM NVP-231 and 4-OI.

(b). The CAT activity in AML12 cells cotreated with 2 μM NVP-231 and 4-OI was measured by the CAT Activity Assay Kit. (n=3/group)

(c). The MDA level in AML12 cells cotreated with 2 μM NVP-231 and 4-OI was measured by the MDA Assay Kit. (n=3/group)

(d). The viability of stable CERK-knockdown AML12 cells treated with 4-OI.

(e). The CAT activity in stable CERK-knockdown AML12 cells treated with 4-OI was measured by the CAT Activity Assay Kit. (n=3/group)

(f). The MDA level in stable CERK-knockdown AML12 cells treated with 4-OI was measured by the MDA Assay Kit. (n=3/group)

one-way ANOVA was used to analyze the significant differences. * $p < 0.05$,** $p <$

0.01, *** $p <$ 0.001, **** $p <$ 0.0001, ns, no significan

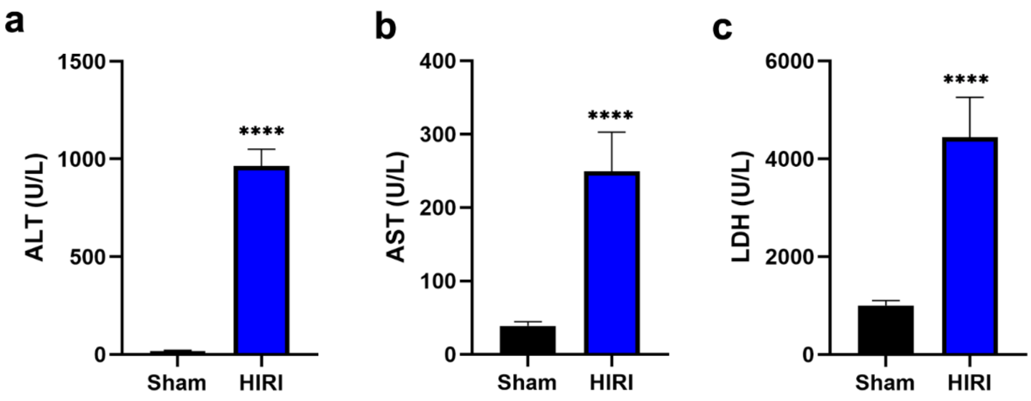

Supplementary Fig. 12. The liver enzymes in mice with HIRI

The serum levels of ALT (a), AST (b), and LDH (c) in mice with HIRI. The number of mice was 5 in each group at each time point. Student t-test was used to analyze the significant differences. **** $p < 0.0001$

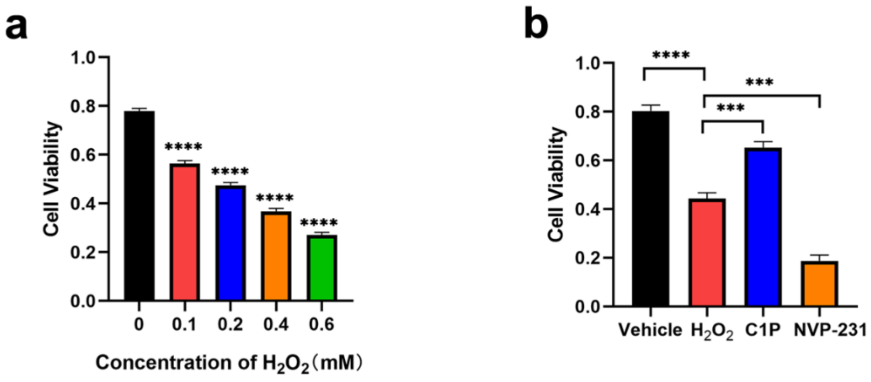

Supplementary Fig. 13. The effects of C1P or NVP-231 for human primary hepatocytes treated with H_2O_2

(a). The viability of human primary hepatocytes treated with different concentrations of H_2O_2 for 24 h to induce oxidative damage. (n=3/group)

(b). The viability of human primary hepatocytes treated with 20 μM C1P or 2 μM NVP-231 for 24 h when exposed to H_2O_2 (0.4 mM). (n=3/group)

one-way ANOVA was used to analyze the significant differences;. ***$p < 0.001$,**** $p < 0.0001$.
